# Comparative Genomic Analysis of Biofilm-Forming Polar *Microbacterium* sp. Strains PAMC22086 and PAMC21962 Isolated from Extreme Habitats

**DOI:** 10.3390/microorganisms11071757

**Published:** 2023-07-05

**Authors:** Byeollee Kim, Saru Gurung, So-Ra Han, Jun-Hyuck Lee, Tae-Jin Oh

**Affiliations:** 1 Department of Life Science and Biochemical Engineering, SunMoon University, Asan 31460, Republic of Korea; luca4163@sunmoon.ac.kr (B.K.); gurungsaru634@gmail.com (S.G.); 2 Bio Big Data-Based Chungnam Smart Clean Research Leader Training Program, SunMoon University, Asan 31460, Republic of Korea; 553sora@hanmail.net; 3 Genome-Based BioIT Convergence Institute, Asan 31460, Republic of Korea; 4 Research Unit of Cryogenic Novel Materials, Korea Polar Research Institute, Incheon 21990, Republic of Korea; junhyucklee@kopri.re.kr; 5 Department of Pharmaceutical Engineering and Biotechnology, SunMoon University, Asan 31460, Republic of Korea

**Keywords:** comparative genomics, polar bacteria, biofilm production, *Microbacterium*, microtiter dish biofilm formation assay

## Abstract

The members of *Microbacterium* isolated from different environments are known to form peptidoglycan. In this study, we compared the biofilm-forming abilities of *Microbacterium* sp. PAMC22086 (PAMC22086), which was isolated from the soil in the South Shetland Islands and *Microbacterium* sp. PAMC21962 (PAMC21962), which was isolated from algae in the South Shetland Islands. The analysis of average nucleotide identity and phylogeny of PAMC22086 revealed a 97% similarity to *Microbacterium oxydans* VIU2A, while PAMC21962 showed a 99.1% similarity to *Microbacterium hominis* SGAir0570. For the comparative genomic analysis of PAMC22086 and PAMC21962, the genes related to biofilm formation were identified using EggNOG and KEGG pathway databases. The genes possessed by both PAMC22086 and PAMC21962 are *cpdA*, *phnB*, *rhlC*, and *glgC*, which regulate virulence, biofilm formation, and multicellular structure. Among the genes indirectly involved in biofilm formation, unlike PAMC21962, PAMC22086 possessed *csrA*, *glgC*, and *glgB*, which are responsible for attachment and glycogen biosynthesis. Additionally, in PAMC22086, additional functional genes *rsmA,* which is involved in mobility and polysaccharide production, and *dksA*, GTPase, and *oxyR,* which play roles in cell cycle and stress response, were identified. In addition, the biofilm-forming ability of the two isolates was examined in vivo using the standard crystal violet staining technique, and morphological differences in the biofilm were investigated. It is evident from the different distribution of biofilm-associated genes between the two strains that the bacteria can survive in different niches by employing distinct strategies. Both strains exhibit distinct morphologies. PAMC22086 forms a biofilm that attaches to the side, while PAMC21962 indicates growth starting from the center. The biofilm formation-related genes in *Microbacterium* are not well understood. However, it has been observed that *Microbacterium* species form biofilm regardless of the number of genes they possess. Through comparison between different *Microbacterium* species, it was revealed that specific core genes are involved in cell adhesion, which plays a crucial role in biofilm formation. This study provides a comprehensive profile of the Microbacterium genus’s genomic features and a preliminary understanding of biofilm in this genus, laying the foundation for further research.

## 1. Introduction

Since being first described in 1919, there have been 155 species of the genus *Microbacterium* with validly published names according to the List of Prokaryotic Names with Standing in Nomenclature website (www.bacterio.cict.fr/index.html accessed on 28 June 2023). The genus *Microbacterium*, which is known for gram-positive, asporogenous, and rod-shaped bacteria, has been isolated from diverse habitats, including soil [1], air [2], sediment [3], plants [4], feces [5], contaminated areas [6], compost [7], desert areas [8], and the gut [9]. Recently, a new species has been discovered to have diaminobutyric acid on peptidoglycan, and studies on its structural diversity and biofilm generation in *Microbacterium* have been reported [10,11,12,13,14,15,16].

Biofilms in bacteria naturally provide a protective mechanism against stress. As per the definition by Donlan et al. [16], biofilm formation occurs when complex microbial ecosystems irreversibly adhere and grow on a surface, where they produce exopolysaccharides (EPS), eDNA, as well as proteins, which together form the extracellular matrix [17]. The biofilm matrix protects microorganisms from host defenses and antibiotics, ultimately helping them survive or spread within the host [18,19]. During biofilm development, bacteria adhere to the surface both reversibly and irreversibly. Based on previous studies on *Pseudomonas aeruginosa*, which is one of the most studied bacteria associated with biofilm formation, the presence of the most common *lasR* and *rhlR* genes are known to be regulated by multiple genes such as *qscR*, *lasl*, *lasB*, *pqsR*, *pqsABCDH*, *oprI*, *oprL*, *algD*, *gyrB*, *toxA*, *ecfX*, *eta*, and *fliC* [20,21]. Although biofilms in bacteria have been studied extensively, a combined study of biofilm experiments and genome analysis concerning *Microbacterium* has not yet been published. To the best of our knowledge, *Microbacterium* has been reported to possess the ability to produce biofilms, and a few genes involved in biofilm production have been identified [17,22]. According to Wagner et al. (2021), studying bacteria from various environments is necessary to explain their diversity and association with genes and biofilm formation [23].

Hence, in this study, we aimed to (i) conduct genomic analysis of *Microbacterium* isolated from the polar region, (ii) perform phylogenetic analysis and clustering of selected genomes based on active functional groups, (iii) explore the predicted biofilm-forming genes in *Microbacterium* sp. PAMC22086 and *Microbacterium* sp. PAMC21962, and (iv) compare both the *Microbacterium* strains regarding biofilm formation. It is hypothesized that this study will provide new insights into the correlation between genome and biofilm by using genome analysis tools and visualized biofilm experiments.

## 2. Materials and Methods

### 2.1. Bacterial Isolation and Growth Curve Measurements

*Microbacterium* sp. PAMC22086 was isolated from the soil in the South Shetland Islands and *Microbacterium* sp. PAMC21962 was isolated from algae in the South Shetland Islands. These two species were deposited by the Korea Polar Research Institute. We cultured the strains on different types of media: Soy Broth (TSB) and Marine Agar (MA). We selected the seed culture medium demonstrating the best growth for each strain. For seed cultures, TSB was used for PAMC22086 and MA was used for PAMC21962. The frozen bacterial samples were reactivated with a 1% (*v*/*v*) inoculum and cultured for 24 h. To prepare bacterial suspension cultures, we cultured the isolates on media to form single colonies and then cultured them in their respective liquid medium with 200 rpm at 15 °C for 24 h. To confirm the growth curves of PAMC22086 and PAMC21962, we cultured a 5% (*v*/*v*) inoculum of the reactivated strains and added them to their corresponding growth media. To compare the growth curves in regular media and biofilm-forming media (BFM), only the suspension culture was switched to BFM, and growth was measured using the same method employed for the regular culture. Optical density (OD) was monitored at a wavelength of 600 nm using an Epoch Microplate Spectrophotometer, and the data were processed with Gen5 version 2.09. A growth curve was constructed using GraphPad Prism version 8.0.1 (BioTek Instruments Ltd., Winooski, VT, USA).

### 2.2. DNA Extraction and Genome Sequencing

Genomic DNA was extracted and purified from cultures grown in R2A media at 15 °C using the Qiagen (Venlo, The Netherlands) Blood & Tissue kit, following the manufacturer’s recommended protocol for Gram-positive bacteria. The extracted DNA was confirmed using 1% agarose gel electrophoresis. It was then sequenced using the PacBio Sequel single-molecule real-time (SMRT) sequencing technology (Pacific Biosciences, Menlo Park, CA, USA) with SMRTbell library inserts (20 kb) and SMRT cells. Raw sequence data were generated for both PAMC22086 and PAMC21962 and were assembled de novo using the hierarchical genome-assembly process (HGAP v.4) protocol and HGAP4 assembly using SMRT analysis software (ver. 2.3; Pacific Biosciences, https://github.com/pacificbiosciences/SMRT-Analysis accessed on 28 June 2023) [24]. The circular map of PAMC22086 and PAMC21962 was obtained using Artemis [25].

### 2.3. Genome Annotation and Functional Prediction

We annotated the genomes using Prokka [26] and Rapid Annotations using Subsystems Technology [27]. We predicted coding sequences (CDS) using Bakta [28] for cross-checking. Annotated protein sequences were classified into clusters of orthologous groups (COGs) using an EggNOG mapper with the EggNOG database [29,30]. Biofilm-related pathways were analyzed using KAAS and visualized manually. To identify biofilm-related genes, we annotated candidate genes against the NCBI non-redundant nucleotide databases with an E-value threshold of 1 × 10^−10^ using DIAMOND-blast [31,32,33]. We selected target genes according to the biofilm structural database [34] and gathered protein sequences from UNIPROT using the code of the biofilm structural database [35].

### 2.4. Phylogenetic Analysis and Average Nucleotide Identity

Polymerase chain reaction amplification of the 16S rRNA was performed using universal primers 27F and 1392R. Bacterial-specific 16S ribosomal sequencing was carried out by Cosmogenetech (Cosmogenetech Co., Ltd., Seoul, Republic of Korea). The phylogenetic tree of PAMC22086 and PAMC21962 was compared with other *Microbacterium* species using 16S rRNA phylogenetic analysis, where strains with higher similarities were sorted from all *Microbacterium* species registered in NCBI. Sequences were aligned using MUSCLE [36] and MEGA X [37] to construct a neighbor-joining tree with 1000 bootstrap replicates. The genomes of PAMC22086, PAMC21962, and other *Microbacterium* species were compared using fastANI [38], which executes pairwise genome calculations of the average nucleotide identity (ANI) matrix with default parameters, such as 16 k-mer, one thread, 3000 fragment length, and the ‘--matrix’ command was used. To compare the genomes of *Microbacterium* species registered in NCBI, fasta files were retrieved from NCBI GenBank and downloaded.

### 2.5. Biofilm Formation

We selected two BFMs reported by Fu et al. (2020) [39] to be highly conducive to biofilm formation. BFM-A and BFM-B are composed of different ingredients. BFM-A contains 10 mg/L of Bacto tryptone, 5 mg/L of yeast extract, 10 mg/L of NaCl, 10 mg of glycerol, and 6.1617 mg/L of MgSO_4_·7H_2_O. On the other hand, BFM-B contains 15 mg/L of Bacto tryptone, 5 mg/L of Bacto peptone, 5 mg/L of NaCl, 10 mg/L of glycerol, and 6.1617 mg/L of MgSO_4_·7H_2_O. The Microtiter Dish Biofilm Formation Assay was performed using BFM-B, which is the more active of the two BFMs. The suspension culture was allowed to grow in biofilm-forming media (BFM) until the OD_600_ value reached 1.00 at 15 °C in a shaking incubator. The Microtiter Dish Biofilm Formation Assay was followed according to Fu et al. (2020) [39]. A 96-well flat tissue culture plate (SPL Life Sciences Ltd., Pocheon-si, Republic of Korea) was filled with 200 µL of 1% suspension culture of each bacterium and allowed to grow for 6 days at 15 °C. We prepared each tenfold dilution and allowed it to grow in sets of 96-well plates. After 6 days, biofilm formation was measured by crystal violet staining. We measured the optical density at 575 nm using a spectrophotometer to quantify the experiment.

## 3. Results and Discussion

### 3.1. Overall Genome Features in PAMC22086 and PAMC21962

We present follow-up studies on the extremophilic microorganisms, *Microbacterium* sp. PAMC21962 and PAMC22086. In a previous study, we reported that *Microbacterium* sp. PAMC28756 possesses genes related to carotenoid production, and we have conducted further research on Carbohydrate Active Enzymes (CAZymes) in these microorganisms [40,41]. Research on extremophilic *Microbacterium* is limited, but the draft genome sequence of *Microbacterium* sp. LEMMJ01, isolated from Antarctic Ornithogenic Soil, also showed genes related to carotenoids and terpenes [42]. Furthermore, other *Microbacterium* strains have exhibited various activities, such as heavy metal degradation and arsenic resistance [43,44]. Despite the diversity of reported activities of *Microbacterium*, research on extremophilic *Microbacterium* has primarily focused on producing CAZymes and carotenoids.

**Figure 1:**
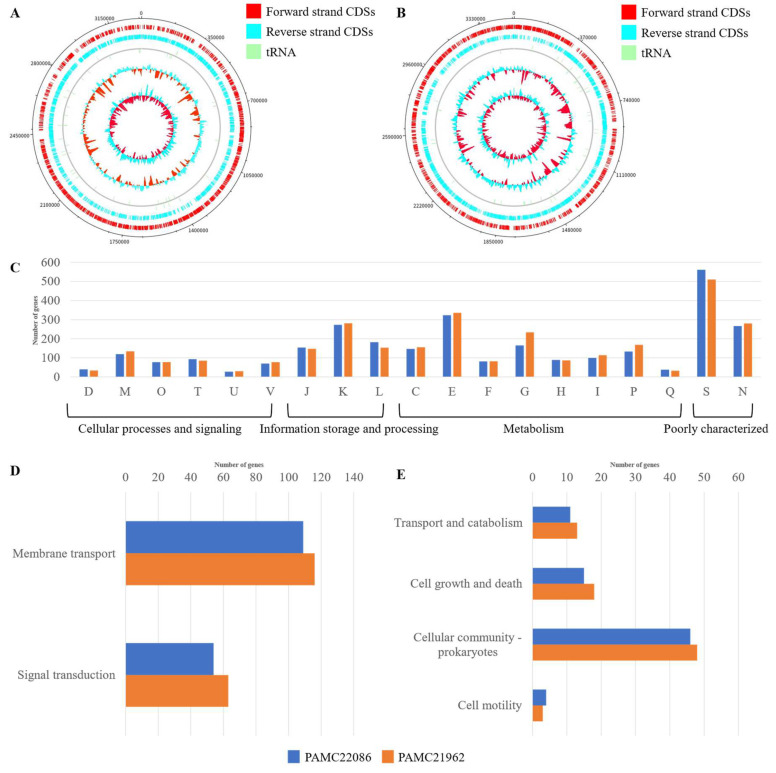
Genome features of *Microbacterium* sp. strains PAMC22086 and PAMC21962. (**A**) Circular map of PAMC22086. (**B**) Circular map of PAMC21962. From the outside to the center: label of genome size, CDSs on the forward strand, CDSs on the reverse strand, tRNA, rRNA, and GC plot, GC skew. (**C**) Comparative analysis of COG for *Microbacterium* sp. strains PAMC22086 and PAMC21962. COG categories; D, Cell cycle control, cell division, and chromosome partitioning; M, Cell wall/membrane/envelope biogenesis; O, Posttranslational modification, protein turnover, and chaperons; T, Signal transduction mechanisms; U, Intracellular trafficking, secretion, and vesicular transport; V, Defense mechanisms; J, Translation, ribosomal structure and biogenesis; K, Transcription; L, Replication, recombination and repair; C, Energy production and conversion; E, Amino acid transport and metabolism; F, Nucleotide transport and metabolism; G, Carbohydrate transport and metabolism; H, Coenzyme transport and metabolism; I, Lipid transport and metabolism; P, Inorganic ion transport and metabolism; Q, Secondary metabolites biosynthesis, transport and catabolism; S, Function unknown; N, Cell motility. (**D**) Membrane transport and signal transduction of KEGG metabolism for *Microbacterium* sp. strains PAMC22086 and PAMC21962. (**E**) Transport and catabolism, cell growth and death, cellular community, and cell motility of KEGG metabolism for *Microbacterium* sp. strains PAMC22086 and PAMC21962. The blue color in (**B**–**D**) represents PAMC22086, while orange represents PAMC21962.

Our study analyzed the complete genome sequences of two *Microbacterium* isolates from polar regions. The complete genome sequence of these strains has been deposited at NCBI (Genbank assembly accession: PAMC22086, GCA_019443525.1; and PAMC21962, GCA_019443465.1). The complete genome of PAMC22086 contains a circular genome of 3,256,707 bases with a 68.2% GC content, whereas PAMC21962 contains a circular genome of 3,047,328 bases with a 71% GC content. Our analysis predicted 3330 and 3328 contigs, respectively. At the same time, eight and four tRNAs were distributed through the genome of PAMC22086 and PAMC21962, respectively (Figure 1A). A total of 3289 genes were predicted for PAMC22086 and 3287 genes were predicted for PAMC21962, as shown in Table 1. In the case of PAMC22086 and PAMC21962, carbohydrate metabolism was identified as the major group of metabolisms represented. On average, both PAMC22086 and PAMC21962 had 510 and 561 COG categories of unknown function, respectively, belonging to the S family (Figure 1C). Most genes were related to amino acid transport and metabolism, transcription, and cell motility, and the compositions were similar to each other. E (Amino acid transport and metabolism), K (Transcription), and T (Signal transduction mechanisms) categories were more prevalent in PAMC22086 than in PAMC21962. On the contrary, PAMC21962 possessed more O (Posttranslational modification, protein turnover, and chaperons), P (Inorganic ion transport and metabolism), and M (Cell wall/membrane/envelope biogenesis) categories than PAMC22086. Among the predicted genes, 1561 (48.90%) from PAMC22086 and 1684 (51.23%) from PAMC21962 were classified into KEGG pathways (Figure 1B). We classified groups of KEGG metabolism as KEGG pathway classification and biofilm-related metabolism of KEGG metabolism as shown in Figure 1D,E, respectively. It was revealed that PAMC21962 had more metabolism than PAMC22086 (data of KEGG metabolism shown in Figure S1).

The strains PAMC22086 and PAMC21962, which were isolated from polar regions, were found to have smaller genomes than the 3.5 Mb size reported in a comparative study of 70 *Microbacterium* genomes, thereby suggesting genomic streamlining for adaptation to the polar environment [43,45]. PAMC28756, previously reported from the polar region, also had a different genome size of 3.5 Mb. In this study, the genome analysis of PAMC22086 and PAMC21962 was expected to provide insights into their advantageous adaptations to the environment.

### 3.2. Phylogenetic Analyses and Average Nucleotide Identity

**Figure 2:**
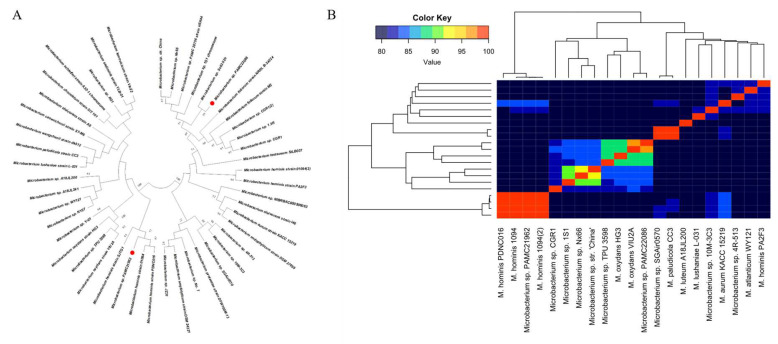
Phylogenetic analysis. (**A**) Phylogenetic tree of 16S rRNA sequence. Our strain is represented by red point in front of species name. (**B**) ANI analysis of 53 *Microbacterium* species. ANI analysis was performed using fastANI and visualized using R. ANI-values ranging from 78% to 100%.

Figure 2A demonstrates PAMC22086 and PAMC21962 cluster with 19 other *Microbacterium* spp. in the phylogenetic tree constructed using the Neighbor-joining method with a p-distance model and a bootstrap value of 1000, with a cutoff value of 50%. The phylogenetic analysis provides an essential picture of the evolutionary relationship between the strains, but it does not provide a direct translation of the overall similarity of genomes. The results of the ANI analysis support the conclusion of the phylogenetic analysis. The ANI approach was used to evaluate the genome similarity between the bacteria, and 21 strains were classified based on their ANI (Figure 2B). For example, PAMC22086 and VIU2A resulted in a higher ANI (97%), thereby suggesting that they belong to the same species as *Microbacterium oxydans* PAMC21962. *Microbacterium hominis* 1094 resulted in a higher ANI (99.93%), indicating that they belong to the same species as *Microbacterium hominis*, similar to strains *Microbacterium* sp. SGAir0570 and *Microbacterium paludicola* CC3, which have a higher ANI (99.1%), suggest that they belong to the species *Microbacterium paludicola* PAMC21962. *Microbacterium hominis* 1094 and *Microbacterium hominis* PDNC016, which are grouped, were identified as strains of *Microbacterium hominisi*, consistent with the results of the phylogenetic analysis. Based on the phylogenetic analyses, our ANI results indicate the presence of errors in the classification of strains *Microbacterium lushaniae* L-031, *Microbacterium atlanticum* WY121, and *Microbacterium luteum* A18JL200. A similar error has also been reported in another genus of bacteria and has generally been corrected through technological advances [46].

Through various genetic tools and comparisons with closely related strains, insight into the association between *Microbacterium* and biofilm-forming ability was gained at both the genetic and experimental levels. Phylogenetic analysis revealed that PAMC22086 is closely related to *Microbacterium oxydans*, a species reported to possess novel Estrone degradation activity, as observed in *Microbacterium oxydans* sp. ML-6 [47]. Regarding PAMC21962, the strain showed a high degree of similarity (99.93%) with *Microbacterium hominis*, a species that has been reported to exhibit activity in degrading 17β-estradiol and has been isolated from human lung aspirates and described in draft genome papers [48,49,50]. Based on our findings, we suggest that further research should be conducted on these species to fully explore their potential for bioremediation applications, thereby highlighting the importance of continued exploration and investigation of these microorganisms.

### 3.3. Identification of Gene Associated with Biofilm Formation

The genes related to biofilm formation identified in PAMC22086 and PAMC21962 are listed in Table 2. We found similar genes, such as *cpdA*, *phnB*, *rhlC*, and *glgC*, in PAMC22086 and PAMC21962. *cpdA* is a phosphodiesterase required for the degradation of cAMP (cyclic adenosine monophosphate), along with two other genes involved in secondary metabolite production, and with two genes in *A. baumannii* for their ability to form these multicellular structures [46]. *phnA*, identified as a part of the pqs operon along with *phnB*, is required to induce genes for the biosynthesis of virulence factors and encodes anthranilate synthase, which is necessary for the biosynthesis of pyocyanin [51,52]. *rhlC* is known as the tolerance of *Pseudomonas* biofilm and virulence factors [53,54]. In heterotrophic bacteria, *glgC* activators are the key metabolites whose presence indicates a high level of carbon and energy in the cell. In contrast, this enzyme’s inhibitors show low metabolic energy. *glgC* is important in glycogen metabolism, and the study suggested that a lack of glycogen elevates swarming, mortality, and biofilm formation [55].

One of the ortholog analyses, EggNOG, was used to manually identify additional genes related to biofilm formation in PAMC22086 and PAMC21962 (Table S1). We manually searched for additional genes such as acetyltransferase genes and ATPase. Acetyltransferase is an enzyme that modifies sugars and plays a crucial role in glycan formation, including lipopolysaccharide and exopolysaccharide. It has also been found to be essential for biofilm formation [69]. ATPases are required for stress tolerance and biofilm formation [70].

A research paper on the complete genome of *Microbacterium* sp. BH-3-3-3 was previously reported concerning biofilms. However, the focus was on its complete genome sequence without providing a detailed description or analysis of its genes or activity [71]. *Microbacterium xylanilyticum* sp. nov, S3-E(T), was isolated from a biofilm [72]. However, it focused on xylan degradation rather than biofilm formation activity. We have identified biofilm-related genes in PAMC22086 and PAMC21962. However, comparative analysis was limited since these genes were not previously reported in *Microbacterium*.

Polar biofilms have become a popular topic in biology due to the discovery of new materials and phenotypes in microorganisms in polar regions. Studies have been conducted to analyze the effects of temperature on these biofilms [55]. In addition, research on the biodiversity of microorganisms in various habitats, including water, epilithic biofilm, cryoconite, mat, ice, sediment, and permafrost soil, has been conducted to understand the microbial ecology of glaciers [73]. Therefore, to better understand polar biofilms, research on this topic should be conducted across various fields and through interdisciplinary collaboration.

### 3.4. Differences in Biofilm-Related Genes between PAMC22086 and PAMC21962

PAMC21962 possesses unique genes, including *varA*, *gacA*, *bcsA*, *lsrR*, *uvrY*, and *pgaC*. The *varA* gene identified in PAMC21962 plays a crucial role in biofilm formation and secretion systems. *varA* has been identified in *Vibrio cholerae* and not in *Microbacterium* [56]. *prrB* RNA, similar to *csrB* RNA in other species, is regulated by *gacA* [74]. *gacA*, also known as *expA* and a homolog of *uvrY* (other orthologs of *uvrY* in *E. coli*, *gacA* in *Pseudomonas*, *varA* in *Vibrio*, and *sirA* in *Salmonella*), is responsible for controlling the expression of many significant virulence genes and is one of the components of regulatory systems involved in biofilm formation in *Streptococcus mutans* [75,76]. In the PAMC21962 isolate, we found *bcsA* (bacterial cellulose synthase), which is generally associated with the synthesis of cellulose and, in association with *bcsB*, forms a protein complex to guide the polymer across the periplasm toward the outer membrane [64].

**Figure 3:**
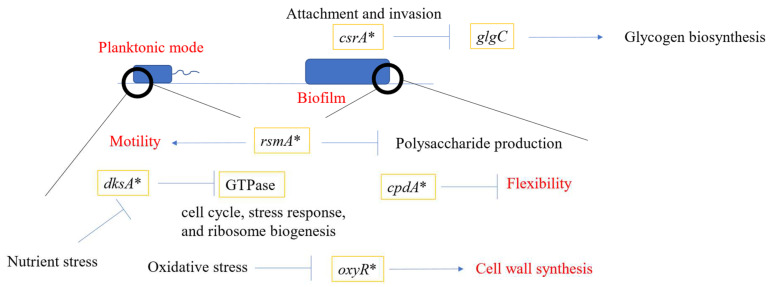
Schematic presentation of biofilm in *Microbacterium* sp. PAMC22086. In planktonic cells, *rsmA* plays a role in motility. The bacteria produced *dksA* and *oxyR* under stress conditions. Three genes were involved in biofilm formation. *cpdA* is known to play a role in flexibility. *csrA* was related to attachment and invasion, which regulates *glgC* and *glgB*. RsmA genes function in polysaccharide production during biofilm-forming steps.

PAMC22086 possesses unique genes, including *csrA*, *rsmA*, *dksA*, and *oxyR*. *csrA* is responsible for attachment and invasion during biofilm formation. A study by Gore et al. in 2010 [77] provided evidence supporting the role of *csrA* in carbon metabolism and virulence. *rsmA* is considered a homologue of *csrA*, as is *rsmE*. It is a global regulatory protein that controls motility, biofilm formation, carbon metabolism, quorum sensing, and virulence functions. We identified *rsmA* in PAMC22086, which suggests that our strain showed visible biofilm since it controls many genes related to biofilm formation. Another gene of interest, *csrA*, a homologue of *rsmA*, was also identified in PAMC22086. It plays a central role in controlling the expression of many major virulence genes [78]. In PAMC22086, we also identified *dksA*, an RNA polymerase-associated transcription factor required to regulate ppGPP (tetraphosphate). When ppGPP forms a complex with RNA polymerase, *dksA* positively or negatively affects transcription as an activator or inhibitor [79]. H_2_O_2_-sensing global regulators (*oxyR*), identified only in the strain PAMC22086, were reported to enhance the extracellular polymeric substances (EPS), which are fundamental constituents of biofilms. *oxyR* effectively regulates oxidative stress defense against H_2_O_2_ in various bacterial species, and these regulatory mechanisms, controlled by *pxyR*, differ depending on the bacterial species. A study carried out by *oxyR* functioning as a repressor regulator of gene expression of PNGI revealed that there are two polysaccharides-producing operons, two poly-*N*-acetyl and K-locus operons, where *oxyR*-controlled expression of two of these loci contributes to the biofilm formation in *A. oleivorans* [69]. The *Pseudomonas* quinolone signal (*pqs*) operon is one of the quorum sensing pathways in *Pseudomonas aeruginosa*, which was found to be present in both strains. We organized and presented biofilm-related genes in PAMC22086 (Figure 3).

Furthermore, we found that some of the genes were related to virulence and virulence factors such as *varA* and *phnA*. *VarA* is one of the genes involved in the Quorem sensing pathway, which is hypothesized to regulate the *luxO* gene, which is finally associated with the expression of virulence genes such as *hapR* [56]. *PhnA* gene encodes for anthranilate synthase, producing anthranilate that acts as a signal for virulence in *Pseudomonas* [59]. Micol et al. (2012) [80] reported that a non-pathogenic *Vibrio* contained at least one potential virulence gene. It remains to be seen whether the identified virulence genes in the *Microbacterium* will exhibit an effect. The genes *cpdA*, *csrA*, *pgaC*, and *varA* are associated with motility and adhesion, both of which are crucial for the formation and growth of biofilms. Unusually, *cpdA* was identified in both species, while only PAMC22086 contained *csrA*. Additionally, PAMC21962 exclusively expressed *pgaC* and *varA*. From a genomic analysis perspective, the unique genes present in each species can be compared to evaluate the virulence of all the identified *Microbacterium* species. Experimental methods can be employed to confirm the pathogenicity of these species. This approach may provide direction for future research on *Microbacterium*. Prediction using artificial intelligence, widely reported recently, is also feasible [81]. Glycogen has been identified in many bacterial species, including strains PAMC22086 and PAMC21962, and has been found to play an important role in transmission, pathogenicity, and environmental viability.

### 3.5. Biofilm Formation Ability of PAMC22086 and PAMC21962

**Figure 4:**
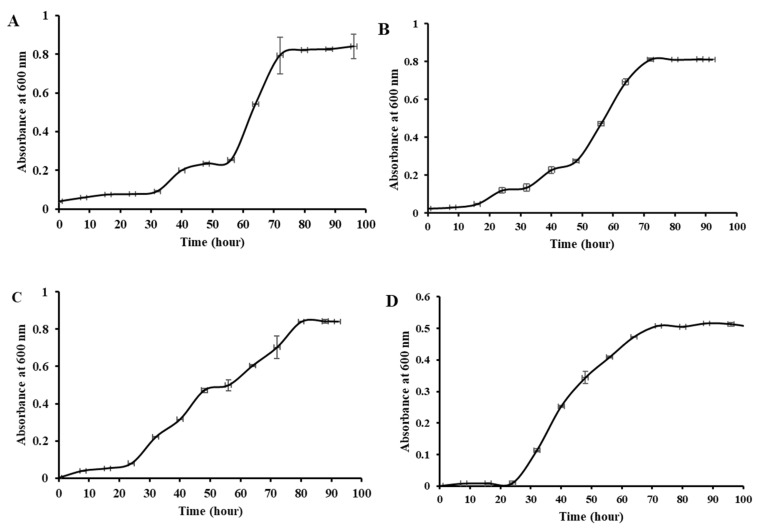
Growth curve. (**A**) PAMC22086 in BFM. (**B**) PAMC21962 in BFM. (**C**) PAMC22086 in TSB. (**D**) PAMC21962 in MA.

In this study, we compared the biofilm-forming ability of *Microbacterium* strains in different biofilm-forming media. Based on the analysis of biofilm forming ability in BFM-A and BFM-B media, the highest biofilm formation was observed in BFM-A (Figure S2). To confirm the biofilm-forming ability of PAMC22086 (closely related to *Microbacterium oxidans*) and PAMC21962 (closely related to *Microbacterium hominis*), we used BFM and TSB for PAMC22086, and BFM and MA for PAMC21962. We allowed the strains to grow for 6 days in each medium, as shown in Figure 4. The biofilm of PAMC22086 was chain-like and mainly distributed at the inner corner of the well, closely adhering to the interface of the bacterial liquid and air. PAMC21962 formed a cluster of microbes at the center of the well, and both strains showed typical three-dimensional biofilm structures compared to the control. However, the matrices produced appeared weak and poorly structured, potentially due to the presence of extracellular polymeric substances (EPSs). After 6 more days of incubation, there was a probable increase in the biofilm clusters with extensive dead cells at the bottom. In the general growth medium, cells appeared to be in a planktonic state, freely floating in the medium. Overall, PAMC22086 produced more rigid and mature biofilm at 12 days, along with matrix development, possibly due to more developed EPSs.

**Figure 5:**
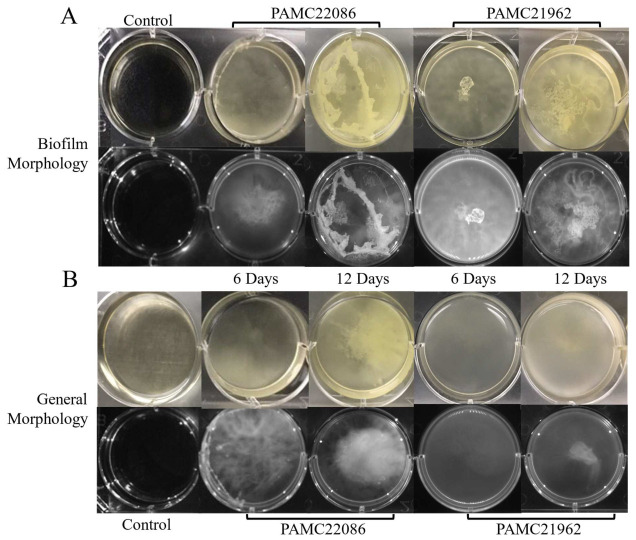
Morphology of *Microbacterium*. (**A**) *Microbacterium* sp. strains PAMC22086 and PAMC21962. (**B**) *Microbacterium* sp. PAMC21962 in biofilm media and planktonic media incubated for 6 days and 12 days.

To investigate the effect of culture media on the growth rate of biofilm formation, we compared the growth curves of the strains in the control and BFM, as shown in Figure 5. Results showed that bacterial growth in BFM increased the growth rate, where the exponential phase was reached in a shorter time interval than the growth in a general growth medium. At almost every observation period after inoculation, the cell density in BFM was higher than in their respective general media. Two different test media were selected based on the method described by Fu et al., 2020 [39], and the TSB system that included glycerol and MgSO4 was the most suitable growth medium for our strain (Figure S2).

**Figure 6:**
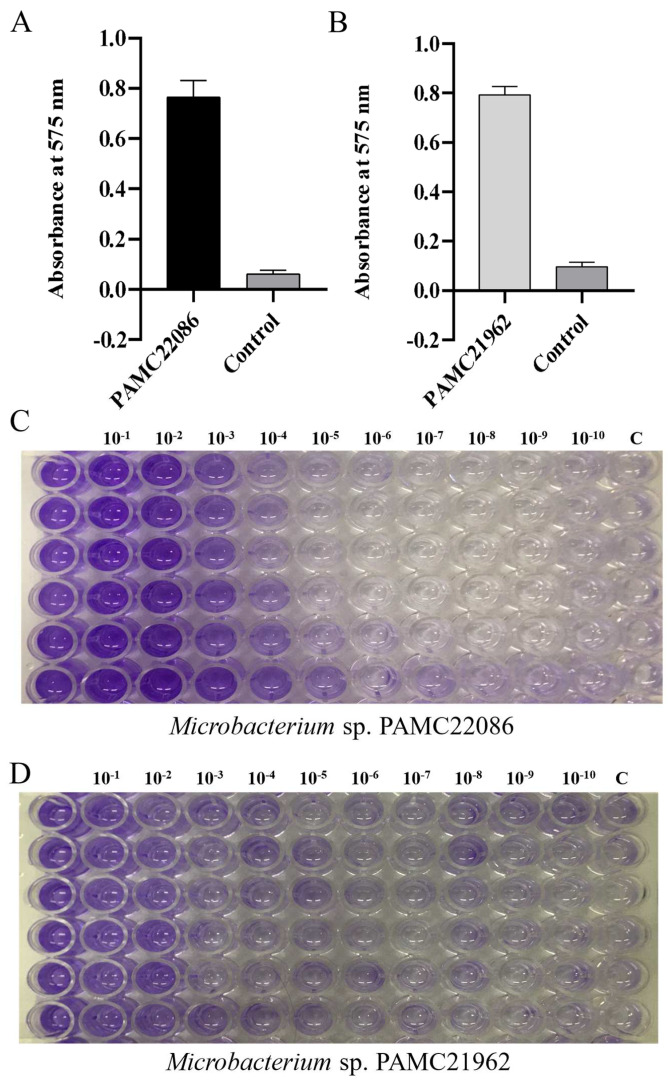
Biofilm stained with crystal violet. (**A**) OD_575_ value of PAMC22086 after 7 days of standing. (**B**) OD_575_ value of PAMC21962 after 7 days of standing. Each experiment was performed at least three times in triplicates. Error bars represent the standard error of mean with SD. (**C**) The stained biofilm of PAMC22086 on the surface of the 96-well plate. (**D**) The stained biofilm of PAMC21962 on the surface of the 96-well plate. The bounded crystal violet dissolved in ethanol.

The biofilm-forming ability in the general growth medium was weak, and visible biofilm could not be observed under the same conditions. To confirm the biofilm-forming ability of the strains, a general biofilm crystal violet assay was performed (Figure 6C,D), and the absorbance measurement results of the bacterial suspension were visualized after 7 days (Figure 6A,B). The OD_575_ value of PAMC22086 in BFM was as high as 0.76, while the control group suspension had an OD_575_ value of only 0.01. Similarly, the OD_575_ value of PAMC21962 was 0.799, while the control group had an OD_575_ value of only 0.018.

In this study, we evaluated the ability of two *Microbacterium* strains derived from polar regions to form biofilms, using morphological differences in biofilm structure and the violet staining method. We also investigated the respective genes related to EPS, tetraphosphate, and oxidative stress defense. Although there are no published papers on the genetic approach in biofilms of *Microbacterium*, several studies have confirmed their biofilm-forming ability. Some studies have reported moderate activity, while *Microbacterium* has shown strong activity in cross-species biofilm comparisons [17,23]. It is hypothesized that comparing the ability to form biofilms in two similar strains isolated from similar environments can provide a good standard for comparing essential elements of *Microbacterium* biofilm formation [22].

## 4. Conclusions

Biofilm formation in *Microbacterium* has been reported rarely, and this is the first study to compare film formation in two microbial species using genome analysis and actual activity. This study demonstrated the biofilm-forming activity of the Microtiter Dish Biofilm Formation Assay and compared the abundance of biofilm-formation genes between PAMC22086 and PAMC21962 through KEGG analysis. *Microbacterium* sp. PAMC22086 and *Microbacterium* sp. PAMC21962 exhibit different biofilm morphologies and types of biofilm-related genes. Our results provide evidence supporting the hypothesis that each strain constitutes a unique microenvironment [82]. Although we compared the genomes and biofilm formation of two *Microbacterium* strains, it is important to note that genes related to biofilm formation in *Microbacterium* are rarely reported, which serves as a limitation. Further research is necessary to understand the precise role and impact of the genes fully. The absence of reported genes directly involved in biofilm formation in *Microbacterium* suggests the necessity for transcriptomic studies in future research. Therefore, it is recommended that future studies should investigate the transcription levels of relevant genes during biofilm formation to determine their activity and significance.

Biofilm formation is an emerging issue related to antibiotic resistance in polar regions. This study utilized genomic analysis of polar microorganisms PAMC22086 and PAMC21962 to screen for enzymes related to biofilm formation. Confirming the biofilm-forming ability of polar microorganisms is crucial for evaluating their activity and enhancing our understanding of their mechanisms of adaptation to the polar environment. Consequently, it is hypothesized that analyzing the biofilm activity of microbial strains derived from polar regions may provide valuable insights into their adaptation to extreme polar environments. This study provides valuable insights into the formation of biofilms in polar microbial strains and comprehends the molecular mechanisms involved in this process.

## Figures and Tables

**Table 1:** Genomic features of *Microbacterium* sp. strains PAMC22086 and PAMC21962.

General Features	PAMC21962	PAMC22086
Genome Size (bp)	3,047,328	3,256,707
Contig (bp)	3328	3330
N50 length	3,509,180	3,311,678
GC%	71	68.2
CDSs	3079	3070
Number of proteins	3205	3077
Number of tRNA genes	46	47
Number of rRNA genes	6	6

**Table 2:** Distribution of biofilm-related genes from PAMC22086 and PAMC21962.

Gene	Genes of PAMC21962 and PAMC22086	Origin	Function	Reference
*varA*	PAMC21962.peg.1943	*Vibrio cholerae*	*varA* controls the expression of numerous genes, most notably those required for virulence.	[56]
*csrA*	PAMC22086.peg.3288	*Escherichia coli*	The *csrA* activates biofilm dispersal under various conditions.	[57]
*cpdA*	PAMC21962.peg.3315 PAMC22086.peg.2094	*Pseudomonas aeruginosa*	The *cpdA* is required for cAMP homeostasis and regulation of virulence factors.	[58]
*phnA*	PAMC21962.peg.540	*Pseudomonas aeruginosa*	The *phnA* and *phnB* act as a signal to modulate biofilm formation and virulence.	[59]
*phnB*	PAMC21962.peg.2458 PAMC22086.peg.645	*Pseudomonas aeruginosa*		[60]
*rhlC*	PAMC21962.peg.3145 PAMC22086.peg.1909	*Pseudomonas aeruginosa*	The function of *rhlC* is to act as a ‘biofilm shield,’ significantly contributing to the increased tolerance of *P. aeruginosa* biofilms.	[54]
*gacA*	PAMC21962.peg.1943	*Pseudomonas aeruginosa*	The *gacA* is a positive regulator of the production of the autoinducer N-butyryl-homoserine lactone and the formation of the virulence factors, such as pyocyanin.	[61]
*rsmA*	PAMC22086.peg.3288	*Xanthomonas campestris*	The *rsmA* regulates biofilm formation in *Xanthomonas campestris* through a regulatory network that involves cyclic di-GMP and the Clp transcription factor.	[62]
*dksA*	PAMC22086.peg.550	*Pseudomonas aeruginosa*	The *dksA* regulates virulence gene expression.	[63]
*bcsA*	PAMC21962.peg.2698	*Rhodobacter sphaeroides*	The *bcsA* is a cellulose synthase that encodes cellulose synthesis, an essential component of biofilms.	[64]
*lsrR*	PAMC21962.peg.2174	*Escherichia coli*	The *lsrR* regulates the uptake of AI-2, which binds with *lsrR* to mediate biofilm architecture and formation by coordinating the interactions of genes related to biofilm formation.	[65]
*uvrY*	PAMC21962.peg.1943	*Escherichia coli*	The *uvrY* expression of type 1 fimbriae, an important adhesin that facilitates adhesion to various abiotic surfaces.	[66]
*glgC*	PAMC21962.peg.285 PAMC22086.peg.2598	*Escherichia coli*	The *glgBXCAP* operon influences physiological activities such as growth rate, glycogen accumulation and structure, biofilm formation, and environmental stress endurance.	[52]
*glgP*	PAMC21962.peg.158	*Escherichia coli*		
*pgaC*	PAMC21962.peg.2862	*Klebsiella pneumoniae*	The *pgaC* regulates the production of Poly-N-acetylglucosamine, which plays a crucial role in biofilm formation.	[67]
*oxyR*	PAMC22086.peg.2252	*Vibrio parahaemolyticus*	The *oxyR* participates in pathogenesis by oxidative stress defense mechanism and promoting biofilm formation.	[68]

## Data Availability

The complete genome sequence was deposited in the GenBank database under the assembly number PAMC22086, GCA_019443525.1; PAMC21962, GCA_019443465.1.

## Supplementary Materials

The following supporting information can be downloaded at: https://www.mdpi.com/article/10.3390/microorganisms11071757/s1, Figure S1: Comparative analysis of KEGG metabolism for *Microbacterium* sp. Strains PAMC22086 and PAMC21962. Figure S2: Biofilm-forming ability in three types of media. Table S1: Putative biofilm-related genes identified in PAMC21962 and PAMC22086.
