# Supplementary material for: Comparative Genomic Analysis of Biofilm-Forming Polar Microbacterium sp. Strains PAMC22086 and PAMC21962 Isolated from Extreme Habitats
Source: Microorganisms. 2023 Jul 5;11(7):1757. doi: 10.3390/microorganisms11071757 (PMC10384088; doi:10.3390/microorganisms11071757)
Supplement: Supplementary file 1 [file microorganisms-11-01757-s001.zip › microorganisms-2419092-supplementary/microorganisms-2419092-Supplementary2.pdf]

Comparative Genomic Analysis of Biofilm-forming Polar *Microbacterium* sp. Strains PAMC22086 and PAMC21962 Isolated from Extreme Habitats

Byeollee Kim[1†], Saru Gurung[1†], So-Ra Han[2,3], Jun Hyuck Lee[4], and Tae-Jin Oh[1,2,3,5*]

[1] Department of Life Science and Biochemical Engineering, SunMoon University, Asan 31460, Republic of Korea.

[2] Bio Big Data-based Chungnam Smart Clean Research Leader Training Program, SunMoon University, Asan 31460, Republic of Korea.

[3] Genome-based BioIT Convergence Institute, Asan 31460, Republic of Korea.

[4] Research Unit of Cryogenic Novel Materials, Korea Polar Research Institute, Incheon 21990, Republic of Korea.

[5] Department of Pharmaceutical Engineering and Biotechnology, SunMoon University, Asan 31460, Republic of Korea.

*Correspondence: Prof. Tae-Jin Oh

†These authors contributed equally to this work.

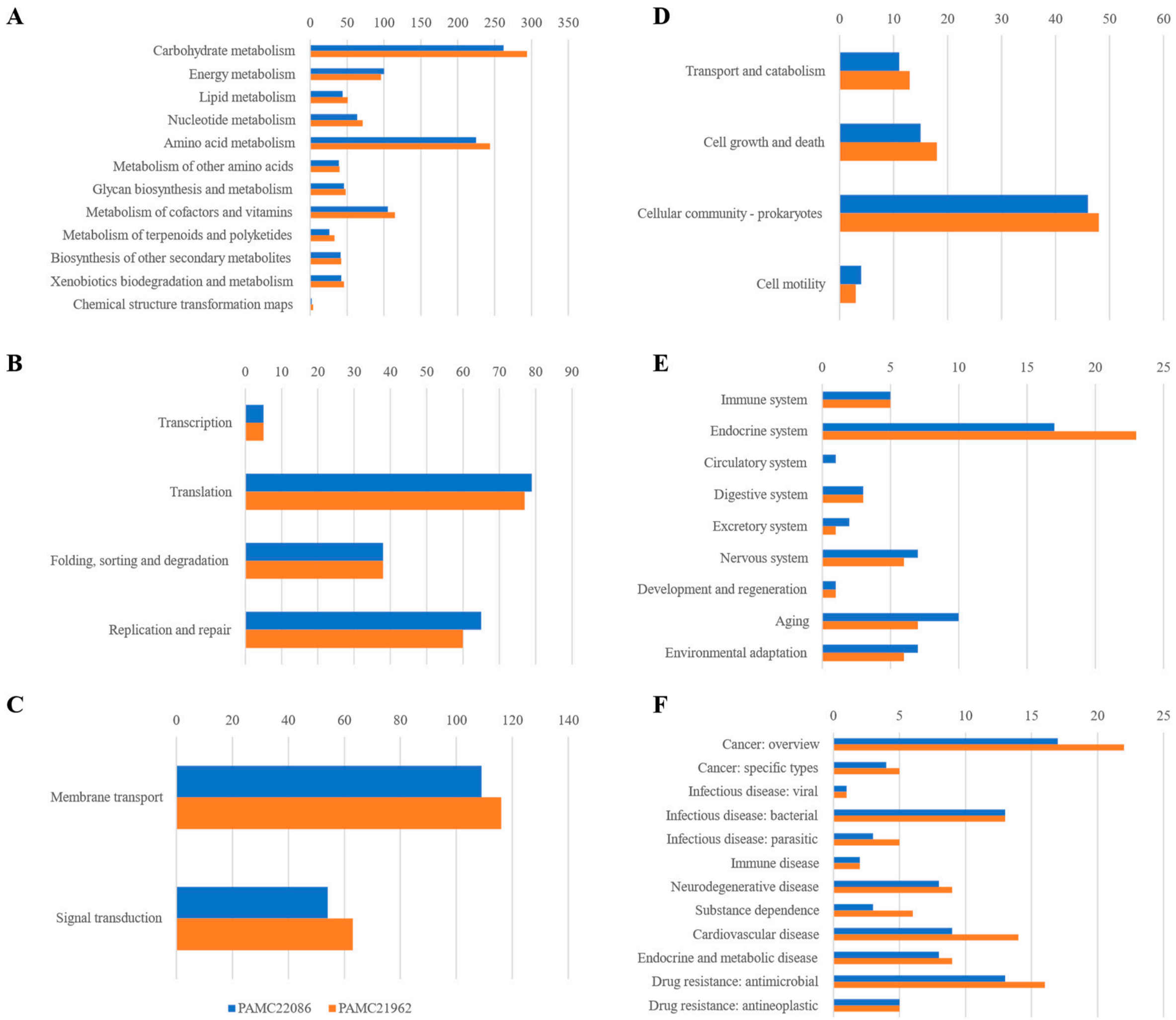

Figure S1. Comparative analysis of KEGG metabolism for *Microbacterium* sp. strains PAMC22086 and PAMC21962. (a) Metabolism. (b) Genetic information processing. (c) Environmental information processing. (d) Cellular processes. (e) Organismal systems. (f) Drug development.

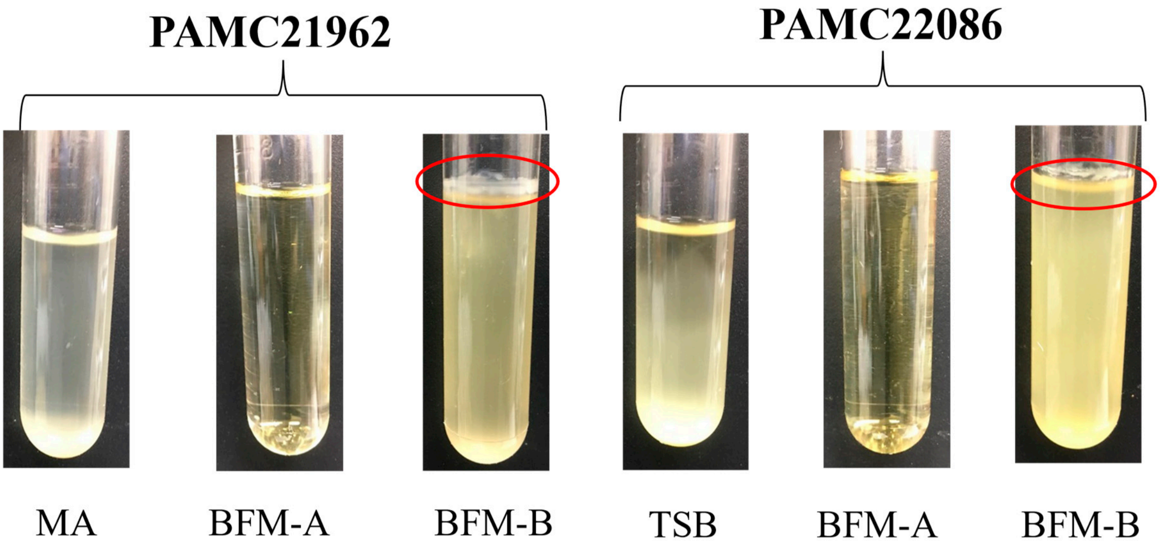

Figure S2. Biofilm-forming ability in three types of media. MA, Marine Agar; TSB, Soy Broth; and BFM-A and BFM-B media are biofilm-forming media. The component of BFM-A and BFM-B is mentioned in section 2.1.
